# Platelet-Derived Growth Factor-D Activates Complement System to Propagate Macrophage Polarization and Neovascularization

**DOI:** 10.3389/fcell.2021.686886

**Published:** 2021-06-02

**Authors:** Zhen Xiong, Qianqian Wang, Wanhong Li, Lijuan Huang, Jianing Zhang, Juanhua Zhu, Bingbing Xie, Shasha Wang, Haiqing Kuang, Xianchai Lin, Chunsik Lee, Anil Kumar, Xuri Li

**Affiliations:** State Key Laboratory of Ophthalmology, Zhongshan Ophthalmic Center, Sun Yat-sen University, Guangzhou, China

**Keywords:** PDGF-D, C1qa, C3, macrophage polarization, inflammation

## Abstract

Platelet-derived growth factor-D (PDGF-D) is highly expressed in immune cells. However, the potential role of PDGF-D in immune system remains thus far unclear. Here, we reveal a novel function of PDGF-D in activating both classical and alternative complement pathways that markedly increase chemokine and cytokine responses to promote macrophage polarization. Pharmacological targeting of the complement C3a receptor using SB290157 alleviated PDGF-D-induced neuroinflammation by blocking macrophage polarization and inhibited pathological choroidal neovascularization. Our study thus suggests that therapeutic strategies targeting both PDGF-D and the complement system may open up new possibilities for the treatment of neovascular diseases.

## Introduction

Tissue inflammation is a cellular response initiated by various factors, such as invasion of foreign material and microbes and clearance of damaged cellular debris to maintain tissue homeostasis (Medzhitov, 2008). Low levels of inflammatory responses also help maintain normal homeostasis (Chen and Xu, 2015). Dysfunction or hyperactivation of inflammation results in various inflammatory neurodegenerative disorders, such as age-related macular degeneration (AMD), Alzheimer’s disease, Parkinson’s disease, and uveitis (Tan et al., 2020; Yang et al., 2020). AMD remains a significant cause for progressive loss of central vision, if uncontrolled, leading to legal blindness (DeAngelis et al., 2017). Microglia, resident immune cells in the retina, choroidal macrophages (Yu et al., 2020) and retinal pigment epithelial (RPE) cells play a central role during inflammation by secreting various chemokines, cytokines, growth factors and elements of the complement system (Holtkamp et al., 2001; Shi et al., 2008; de Oliveira Dias et al., 2011). Additionally, several studies have identified alterations of the complement pathway in AMD pathogenesis (Wu and Sun, 2019). Small menagerie of complement proteins can activate the complement system via classical, lectin and alternative pathways, converging on the critical complement component C3, to generate C3a, C5a and membrane-attacking complex (MAC) C5b-C9, acknowledged in the drusen of AMD patients (Crabb et al., 2002; Mullins et al., 2014; Kim et al., 2020). Increasing evidence suggests that persisting inflammatory milieu supports the classical macrophage activation (M1 polarization) to generate tissue-destructing proinflammatory signals, and alternative macrophage activation (M2 polarization) generates anti-inflammatory signals to promote pathological angiogenesis (Apte, 2010). However, currently, it is not well understood how the complement system in CNV is activated, although its components are found in neovascular lesions of wet AMD patients.

Platelet-derived growth factor-D (PDGF-D), a member of the PDGF family, has been shown to exert diverse functions under physiological and pathological conditions (Bergsten et al., 2001; Kazlauskas, 2017; Folestad et al., 2018; Kumar and Li, 2018). Several studies have implicated PDGF-D in the promotion of inflammation and in the increased migration of monocytes and macrophages under pathological conditions. During intracerebral hemorrhage, PDGF-D promotes neuroinflammation and enhances macrophage infiltration (Yang et al., 2016). Adipocyte-derived PDGF-D promotes adventitial fibrosis with inflammation, thereby contributing to the development of aortic aneurysm in obesity (Zhang et al., 2018). Ostendorf et al. (2006) and Boor et al. (2007) demonstrated that the administration of human mAb CR002, a PDGF-D antibody, reduced glomerular infiltration of monocytes/macrophages and prevented epithelial-mesenchymal transition. In our previous work, we have shown that *Pdgfd* knockdown by shRNA inhibited the macrophage infiltration and reduced choroidal neovascularization (Kumar et al., 2010). These studies highlight the effects of PDGF-D on inflammatory cell infiltration under pathological conditions. However, the mechanism by which PDGF-D induces inflammatory cell activation and migration is not well understood. Notably, a recent study has shown that PDGF-D inhibits tumor growth by binding to natural killer (NK) cell receptor NKp44, leading to the production of tumor-suppressive cytokines by NK cells (Barrow et al., 2018), in contrast to many previous reports showing angiogenic and oncogenic effects of PDGF-D (Li et al., 2003; Kumar et al., 2010; Kumar and Li, 2018). Thus, more in-depth studies are warranted to verify the functions of PDGF-D and the underlying mechanisms.

## Materials and Methods

### Animals

All animal experiments were approved by the Animal Use and Care Committee of Zhongshan Ophthalmic Center at the Sun Yat-sen University, Guangzhou, People’s Republic of China. C57BL/6J (6–8 weeks old) mice were purchased from Pengyue Company (Shandong, China). All mice were maintained on a 12-h light/dark cycle with water and chow provided *ad libitum* and were housed in an SPF facility in the Ophthalmic Animal Laboratory of Zhongshan Ophthalmic Center at the Sun Yat-sen University. Five minutes after intraperitoneal injection of 4% chloral hydrate (10 ml/kg body weight), mice were anesthetized before treatment or euthanized directly by cervical dislocation.

### Cell Culture

RAW264.7 cells (Zhong Qiao Xin Zhou Biotechnology Co., Ltd., China, cat: ZQ0098) were cultured in the Dulbecco’s modified Eagle’s medium (DMEM) (Corning, cat: 10–013–CV) supplemented with 10% heat inactivated FBS (ExCell Bio, China, cat: FSS050) and 1% penicillin/streptomycin (Corning, cat: 30-002-Cl). THP-1 cells (Zhong Qiao Xin Zhou Biotechnology Co., Ltd, China, cat: ZQ0086) were cultured in RPMI-1640 medium (Corning, cat: 10-040-CV) supplemented with 10% heat inactivated FBS and 1% penicillin/streptomycin as mentioned above. THP-1 cells were differentiated (dTHP1) by incubation with 150 nM phorbol-12-myristate-13-acetate (PMA) (Sigma, cat: P8139) in complete medium for 24 h followed by 24 h PMA-free and serum-free medium treatment to reduce cell detachment (Spano et al., 2013).

### Preparation of Conditioned Medium

Primary human retinal pigment epithelial (HRPE) cells (Sciencell, cat: 6540) at passage 5 were used for collecting the conditioned medium (CM). Briefly, HRPE cells were cultured in epithelial cell medium (EpiCM, Sciencell, cat: 4101) containing 10% FBS (Sciencell, cat: 0010), 1% epithelial cell growth supplement (EpiCGS, Sciencell, cat: 4152) and 1% penicillin/streptomycin (P/S, Sciencell, cat: 0503). After reaching 80–90% confluence HRPE cells were starved in EpiCM without any supplements for 12 h, followed by 12 h treatment with/without 50 ng/ml recombinant human PDGF-D protein (R&D, cat: 1159-SB/CF). Cells were rinsed and cultured in the supplement-free Dulbecco’s modified Eagle Medium / Ham’s F-12 Mix (DMEM/F-12) (Corning, cat:10-092-CV) and after 24 h collected medium was filtered through 0.22 μm filter (Millex^TM^ GP Filter Unit, Millipore, cat: SLGP033NB), and stored at –80°C until use.

### Proliferation Assay

RAW264.7 and dTHP1 cell proliferation assays were performed using a Cell Counting Kit-8 assay (CCK8, Dojindo, Japan). Cells were seeded in 96-well culture plates (5,000 cells/well for RAW264.7 cells and 10,000 cells/well for dTHP1 cells, respectively) and 450 nm absorption values were recorded after treatment with the CCK-8 reagent.

### Migration Assay

RAW264.7 cells were seeded in the cell culture inserts (Ibidi, Germany, cat: 80209) at 1 × 10^5^ cells/chamber and the inserts were removed after 12 h to generate cell-free gaps. dTHP1 cells were seeded in 48-well plates (3 × 10^5^ cells/well) and the cell-free area was produced by scratching the wells with a 200 μl pipette tip. Images were obtained at 0 and 24 h, respectively, after treatment and were analyzed using the ImageJ software.

### Construction of Adeno-Associated Virus (AAV) for RPE-Specific PDGF-D Overexpression

The CMV promoter of the AAV vector pAV-CMV-C-FH (Vigenebio, China, cat: pAV100001–OE) was replaced with the human *VMD2* promoter (–598 bp upstream to 378 bp downstream of the transcription start site), known to drive efficient and specific transgene expression in RPE (Alexander and Hauswirth, 2008). The multi-cloning site of the vector was replaced by the coding sequence of human *PDGFD* gene (NM_025208). The AAV was packaged by Vigene Biosciences (Shandong, China) and stored at –80°C.

### Subretinal AAV Injection in Mice

Mouse pupils were dilated by topical application of tropicamide. An intraperitoneal injection of 4% chloral hydrate (10 ml/kg body weight) was performed for anesthesia. Topical anesthesia was performed on the cornea using procainamide. Carboxymethyl cellulose sodium was used to avoid the development of cornea xerosis. Subretinal injection was performed using a sterilized 5 μl syringe (Hamilton, cat: 7633-01) with a 33-gauge blunt needle (Hamilton, cat: 7803-05, 33/15mm/3) through a puncture hole of 0.2 mm in diameter behind the cornea limbus, and AAV (1 μl/eye, 5 × 10^13^ vg/ml) was injected. A successful subretinal injection was indicated by the visualization of the semicircular retinal detachment around the injection site under a microscope or by fundus imaging.

### Intraperitoneal Injection of C3a Antagonist

The 20 mg/ml stock solution of the C3a antagonist SB290157 (MCE, cat: HY-101502A) was prepared by dissolving the compound in a minimal volume of sterile dimethyl sulfoxide (DMSO, Sigma, cat: D4540). SB290157 was further diluted using corn oil to a final concentration of 2 mg/ml for injection at a dose of 30 mg/kg body weight. Three weeks after AAV injection, SB290157 or vehicle was injected intraperitoneally every 2 days for 3 times. In the laser-induced CNV model, the antagonist was intraperitoneally injected 3 times after laser photocoagulation.

### Immunofluorescence Tissue Staining

Sections (10-μm thick) of eyeballs were incubated in 0.5% Triton^TM^ X–100 (Sigma, X100) prepared in phosphate buffered saline for 15 min for permeabilization and then blocked using 5% normal goat serum for 1 h followed by overnight incubation with the primary antibody at 4°C. Primary antibodies used were: anti-IA/IE (BD, 562564), anti-C1q (Abcam, ab182451), anti-PDGFRα (CST, 3169), anti-PDGFRβ (CST, 3174), anti-CD16/32 (BD, 553141), anti-CD206 (Bio-Rad, MCA2235GA), anti-IBA1 (WAKO, 019-19741), anti-CD31 (Bio-Rad, MCA2388), anti-NG2 (Millipore, AB5320), and anti-α-SMA (Sigma, A2547). After three washes, slides were incubated for 1h at room temperature with secondary antibodies (Invitrogen) followed by a 10 min DAPI (Sigma, D9542) incubation. Immunostained sections were imaged using the Zeiss LSM710 laser scanning confocal microscope. Images were processed using ZEN 2012 (Zeiss) and quantified using ImageJ.

For analysis of flat-mounted retinas, 1 week after AAV-PDGF-D subretinal injection, SB290157 (MCE, cat: HY-101502A) was injected intraperitoneally (30 mg/kg body weight) every 2 days. After 1 week, the mice were sacrificed, and the eyes harvested and fixed in 4% PFA for the analysis of flat-mounted retinas. The anterior segment of the eye and lens were removed. The retinas were separated from the sclera and flattened on a glass slide and dissected by making four radial cuts. Flat-mounted retinas were stained with I isolectin B4 (IB4, Thermo Fisher, cat: 121411) and analyzed using a fluorescent microscope AX10 imager.Z2 (ZEISS). The vascular branch points were analyzed using Angio Tool (version 0.5).

### RNA Sequencing and Transcriptomic Analysis

Four weeks after AAV injection, the mice were sacrificed. The eyeballs were removed and the fascial tissues and muscles around the eyeballs dissected on ice. Choroidal tissue was quickly dissected and put into the reagent for RNA isolation. RNA was sent to Shanghai Pharmaceutical Kangde Co., Ltd. (Shanghai, China) for RNA sequencing. Sequenced raw reads were mapped to mouse mm10 reference genome using STAR (v2.4.2a). After alignment, RSEM (V1.2.29) was used to generate FPKM values for known gene models. Differentially expressed genes were identified using DESeq2 (v1.22.2). Fold-changes were estimated according to each sample’s FPKM. Differentially expressed genes were selected using the following filter criteria: *P*-value ≤ 0.1, FDR ≤ 0.1, fold-change ≥ 1.5, mean FPKM ≥ 1. Gene set functional enrichment analysis was performed using cluster Profiler (v3.17.0) with gene set size set to 5–500, *P*-value cutoff set to 0.05, and adjusted *P*-value set to 0.25. Volcano plots were generated using ggplot2 (v3.3.0). Heatmap plots were generated using pheatmap (v1.0.12). Gene-function networks for differentially expressed chemokine genes and related biological processes were visualized using Cytoscape (v3.6.1).

### RNA Isolation and Real-Time Quantitative PCR

Total RNA was isolated using the TRNzol reagent (TIANGEN, cat: DP424) and converted to cDNA using the Fast King RT Kit (TIANGEN, cat: KR116) according to the manufacturer’s instructions. Real-time quantitative PCR was carried out in a 10 μl reaction containing the SYBR Select Master Mix (Vazyme, cat: Q331) in technical quadruplicate using a Quantstudio 6K Flex system (Life Technologies). Results were analyzed using the Quantstudio 12 K Flex Software v1.2.2 (Thermo Fisher Scientific). Relative mRNA levels were calculated based on the 2^−ΔΔCT^ method, using the 18S rRNA as references.

### Protein Extraction and Western Blots

Protein extraction was performed using RIPA lysis buffer with a cocktail of protease and phosphatase inhibitors (Thermo Fisher Scientific, cat: A32961). Lysates were separated using SDS-PAGE under reducing conditions and transferred onto a PVDF membrane (Bio-Rad, cat: 162-0177). Membranes were blocked using 5% defatted milk and immunoblotted with the primary antibodies overnight at 4°C, followed by incubation with the secondary antibodies conjugated with horseradish peroxidase (HRP). The following antibodies were used: anti-PDGFRα (CST, cat: 3169), anti-PDGFRβ (CST, cat: 3174), anti-NRP1 (Abcam, cat: ab81321), anti-PDGF-D (Santa Cruz, cat: sc137030), anti-PDGF-D (R&D, AF1159), anti-C1q (Abcam, cat: ab235454), and anti-C3 (Abcam, cat: 200999). Bands were detected using a Syngene GBOX/CHEMI-XT16 device.

### Choroid Explant Assay

C57BL/6J mice at postnatal day eight were sacrificed, and eyes were enucleated and kept in ice-cold phosphate buffered saline before dissection. After removing the cornea and lens from the anterior of the eye, the peripheral choroid-scleral complex was separated from the retina and cut into pieces (approximately 1 mm × 1 mm). Choroid pieces were transferred into growth factor-reduced Matrigel (BD, Cat: 356231) and seeded in 24-well plates followed by Matrigel solidification for 10 min. A volume of 500 μl of medium was added to each well and incubated at 37°C with 5% CO_2_ for 48 h. The medium was changed every 48 h. Individual explants were imaged daily using an inverted microscope. Areas of choroidal sprouts were quantified using ImageJ.

### Laser Induced CNV

The laser-induced CNV mouse model was performed as described previously (Zhang et al., 2009). Briefly, 8 weeks old female mice were anesthetized by intraperitoneal injection of 4% chloral hydrate (10 ml/kg body weight), and eyes were dilated by topical application of tropicamide. Four laser spots were made by laser photocoagulation (90 mV power, 75 ms duration, 75 μm spot size, Oculight Infrared Laser System 810 nm, IRIDEX Corporation) at an equal distance from the optic nerve in each eye for CNV. The cornea of mice were treated with antibiotic tobramycin ointment locally after laser photocoagulation and mice were placed on a 37°C electric blanket until wake. After 7 days, the eyecups were flat-mounted and the immunohistochemical staining were performed as described (Zhang et al., 2009).

### Statistical Analysis

Gene expression analysis by Q-PCR are expressed as means ± SD. While other results are expressed as means ± SEM. The statistical significance between the control and PDGF-D, or between AAV-GFP and AAV-PDGF-D groups were assessed with the unpaired student’s two-tailed *t* test. Multiple group comparisons were performed with ordinary one-way ANOVA test. Differences between groups were tested with GraphPad Prism software (version 7.04) and considered statistically significant for *P* < 0.05.

## Results

### PDGF-D-Induced Retinal Epithelial Cell Secretome Promotes Macrophage Migration

**FIGURE 1:**
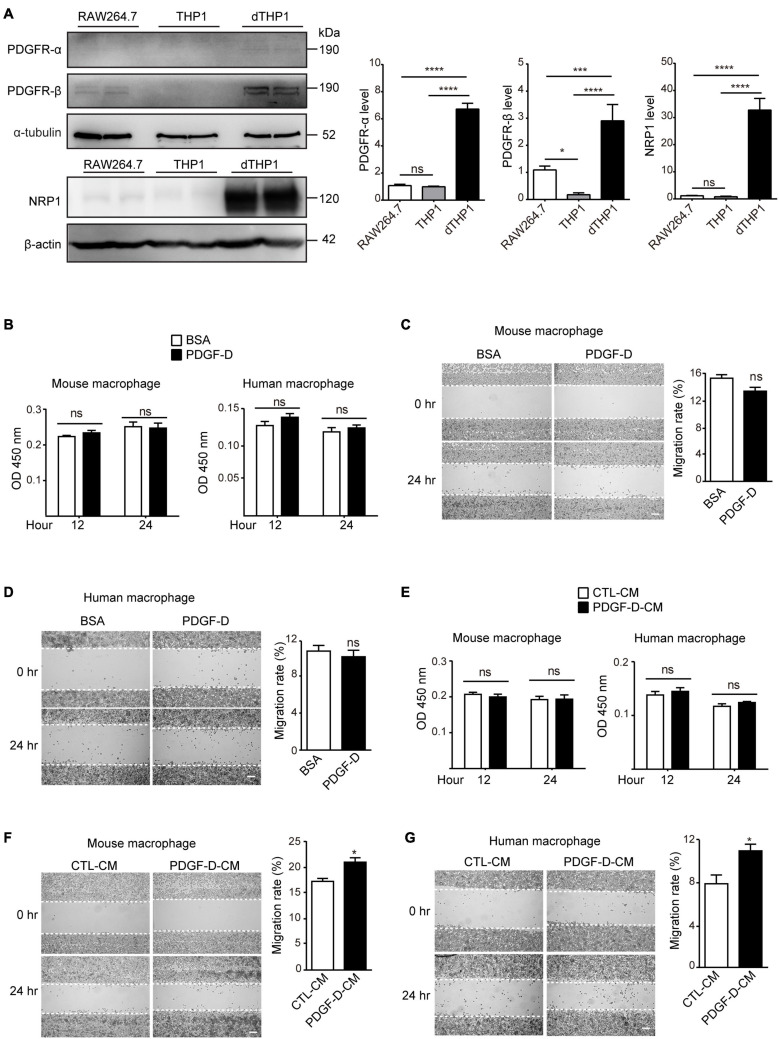
Effect of PDGF-D on macrophage proliferation and migration. **(A)** Immunoblotting showing the expression of PDGFR-α, PDGFR-β and NRP1 in RAW264.7 mouse macrophages, THP1 human monocytes and dTHP1 human macrophages. **(B)** Proliferation of mouse and human macrophages treated with PDGF-D protein for 12 or 24 h. **(C,D)** Migration of mouse **(C)** and human **(D)** macrophages stimulated with PDGF-D protein for 24 h. **(E)** Proliferation of mouse and human macrophages treated with conditioned medium from PDGF-D-treated HRPE cells (PDGF-D-CM). Conditioned medium from HRPE cells without PDGF-D treatment (CTL-CM) was used as a control. **(F,G)** Migration of mouse **(F)** and human **(G)** macrophages treated with conditioned medium from PDGF-D-treated HRPE cells (PDGF-D-CM). Conditioned medium from HRPE cells without PDGF-D treatment (CTL-CM) was used as a control. Scale bars: 400 μm. All the experiments were performed in triplicates. Unpaired two-tailed Student’s *t*-test was used for statistical analysis. *p < 0.05, ***p < 0.001, ****p < 0.0001, ns: not significant.

Under pathological conditions, PDGF-D has been shown to promote macrophage migration (Uutela et al., 2004). However, the mechanism by which PDGF-D promotes macrophage migration is not well understood. To address this, we stimulated murine macrophages (RAW264.7) and differentiated human macrophages (dTHP1) with PDGF-D. Murine macrophages expressed PDGFR-β and the PDGF-D co-receptor NRP1 but not PDGFR-α (Figure 1A and Supplementary Figure 1A). The human monocytic cell line (THP1) did not express PDGF receptors (Figure 1A and Supplementary Figure 1B). However, upon differentiation to macrophages by PMA, they expressed PDGFR-α, PDGFR-β and NRP1 (Figure 1A and Supplementary Figure 1B). PDGF-D stimulation did not promote proliferation (Figure 1B and Supplementary Figures 1C,D) or migration (Figures 1C,D and Supplementary Figures 1E,F) of mouse or human macrophages. Human retinal pigment epithelial (HRPE) cells play a crucial role in the pathophysiology of AMD, and PDGF-D has been shown to promote proliferation and migration of RPE cells (Li et al., 2007). PDGF-D activated the PDGFR-β on HRPE (Supplementary Figure 2A), and conditioned medium (CM) from cultured HRPE cells treated with PDGF-D (PDGF-D-CM) did not affect human or mouse macrophage proliferation (Figure 1E and Supplementary Figures 1G,H). However, and noteworthy, PDGF-D-CM significantly promoted migration of both types of macrophages (Figures 1F,G and Supplementary Figures 2B,C). Hence, PDGF-D-induced HRPE secretome promoted macrophage migration, while PDGF-D did not show a direct effect on them.

### PDGF-D Overexpression in Mouse Retinal Epithelial Cells

**FIGURE 2:**
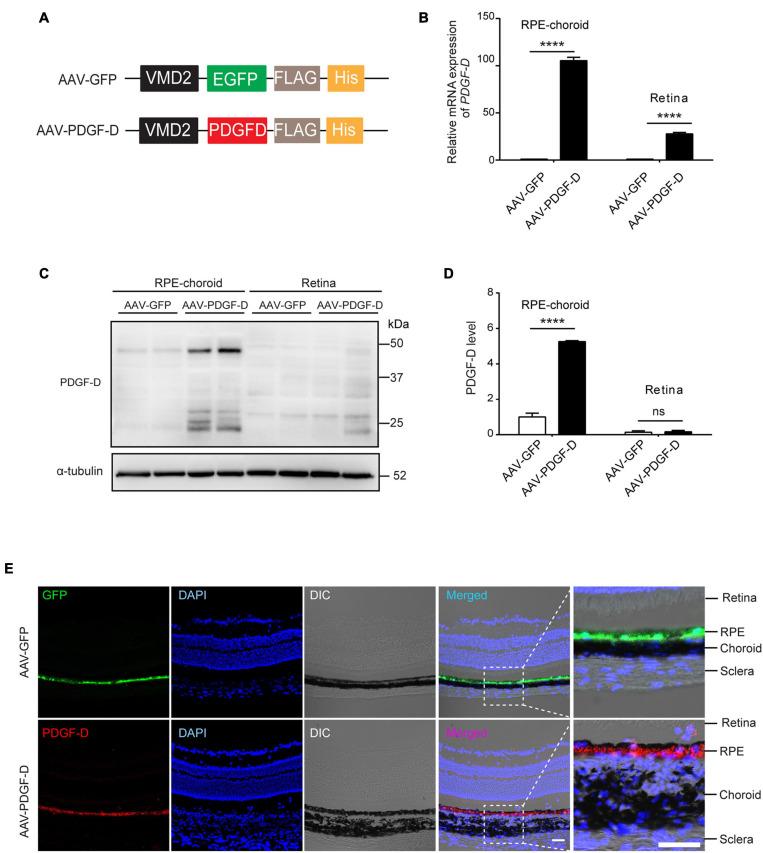
RPE-specific overexpression of PDGF-D *in vivo.* **(A)** Schematic diagram illustrating the AAV vector carrying GFP (AAV-GFP) or human *PDGF-D* (AAV-PDGF-D) gene and RPE specific *VDM2* promoter. **(B)** Real-time PCR results of relative mRNA expression of *PDGF-D* in retinae or RPE-choroid complex from mice injected with AAV-GFP or AAV-PDGF- D for 4 weeks. **(C)** Representative immunoblotting showing PDGF-D expression in mouse retinae or RPE-choroid complex. **(D)** Quantifications of PDGF-D protein levels in **(C)**. **(E)** Immunofluorescence images highlighting RPE-specific expression of GFP or PDGF-D in mouse RPE-choroid complex with 4 weeks AAV-GFP or AAV-PDGF-D injection, respectively. Scale bar: 50 μm. *n* = 5, ****p < 0.0001, ns: not significant.

Recombinant adeno-associated virus (AAV) has been shown to be effective for retinal gene therapy due to their efficient transduction of RPE cells with low toxicity (Vandenberghe and Auricchio, 2012). To over-express PDGF-D in mouse RPE cells, we constructed AAV type 8 (AAV8) vector expressing human PDGF-D (AAV-PDGF-D) driven by the retinal pigment epithelium specific *VMD2* promoter (Figure 2A). PDGF receptors are expressed in both mouse retinae and choroid (Hou et al., 2010; Supplementary Figures 3A–D). Four weeks after subretinal injection of AAV-PDGF-D or AAV-GFP (as a control), overexpression of *PDGF-D* mRNA and protein in the RPE-choroid complex were detected (Figures 2B–D). Moreover, immunofluorescence staining identified PDGF-D or GFP in the RPE layer, respectively (Figure 2E), demonstrating successful RPE-specific PDGF-D overexpression *in vivo*.

### Activation of the Complement Pathway Revealed by Transcriptomic Analysis of PDGF-D-Overexpressing RPE-Choroids

**FIGURE 3:**
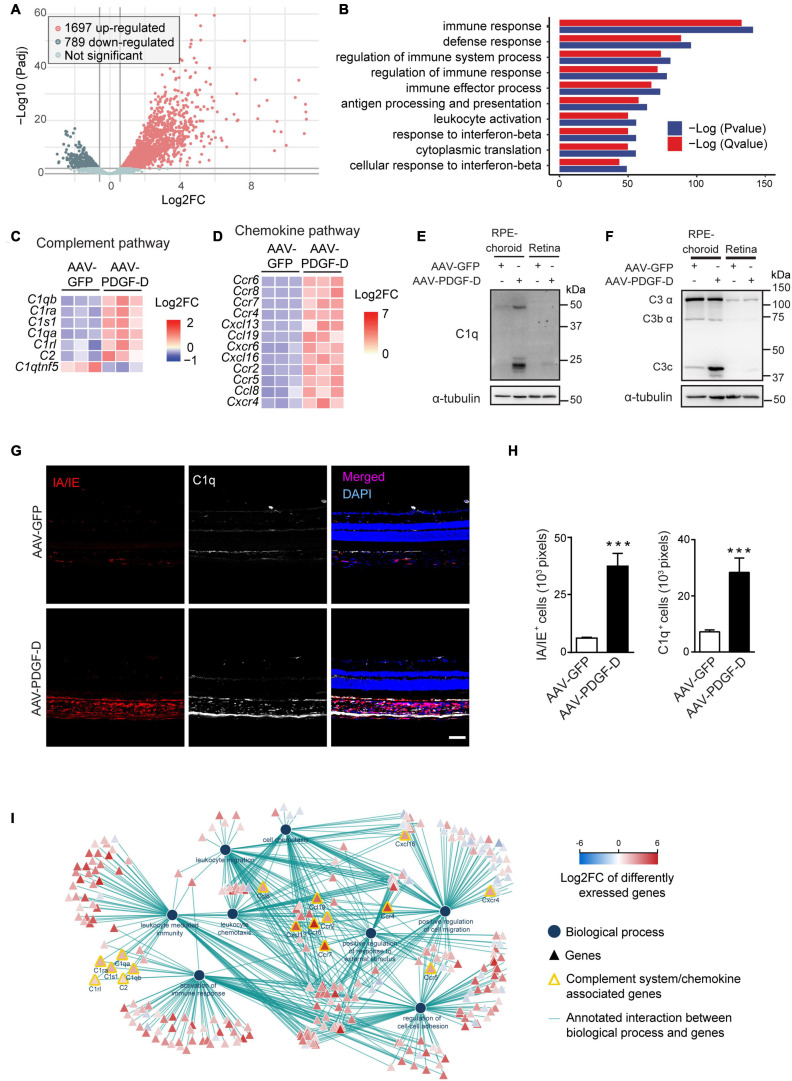
RNA-seq and transcriptomic analysis of PDGF-D-induced complement pathway. **(A)** Volcano plot showing the differentially expressed genes (DEFs) from the RNA-seq data. **(B)** Biological function enrichment gene ontology (GO) analysis of DEGs showing the enriched biological processes in mouse PDGF-D-overexpressing RPE-choroids. **(C,D)** Heatmap of the DEGs associated to complement pathway **(C)** and chemokines and chemokine receptors **(D)** in mouse RPE-choroids injected with AAV-GFP or AAV-PDGF-D. **(E,F)** Immunoblot of C1q **(E)** and C3 **(F)** expression in RPE-choroid complex and retinae from mice with AAV-GFP or AAV-PDGF-D injection. **(G)** Immunofluorescence staining showing the IA/IE and C1q expression in mouse retinae and choroids with AAV-GFP or AAV-PDGF-D injection. **(H)** Quantification of IA/IE and C1q expression in **(G)**. **(I)** Functional network analysis of DEGs showing pathways related to activation of the complement system, immune cell migration and activation of immune responses in PDGF-D-overexpressing RPE-choroids. Scale bar: 50 μm. *n* = 5–6, ***p < 0.001.

To identify PDGF-D-induced downstream pathways, we performed unbiased transcriptomic analysis using the PDGF-D-overexpressing RPE-choroid complex. A total of 2,486 differentially expressed genes (DEGs) were identified. Among them, 1697 were up-regulated and 789 were down-regulated (Figure 3A). Biological function enrichment gene ontology analysis showed that the most enriched biological processes were related to the regulation of immune system (Figure 3B), particularly, the complement pathway (Figure 3C). Other pathways included chemokine and its receptor (Figure 3D and Supplementary Figure 4B), cytokine signaling (Supplementary Figures 4C,E) and regulation of extracellular matrix and growth factors (Supplementary Figures 4D,F). Importantly, both gene (Supplementary Figure 4A) and protein analysis confirmed the activation of both classical (Figure 3E) and alternative complement pathways (Figure 3F). Immunofluorescence staining further identified C1q expression in both RPE and choroids of PDGF-D-overexpressing samples (Figure 3G), where PDGF-D overexpression led to increased accumulation of IA/IE^+^ macrophages in the choroids, which were also positive for C1q staining (Figures 3G,H). Further functional network analysis of DEGs demonstrated pathways related to activation of the complement system, immune cell migration and activation of immune responses in PDGF-D-overexpressing RPE-choroids (Figure 3I). These findings thus underscore PDGF-D overexpression-induced inflammation by activating the complement pathway and chemokine/cytokine signaling.

### PDGF-D-Induced Complement Activation Promotes Macrophage Polarization

**FIGURE 4:**
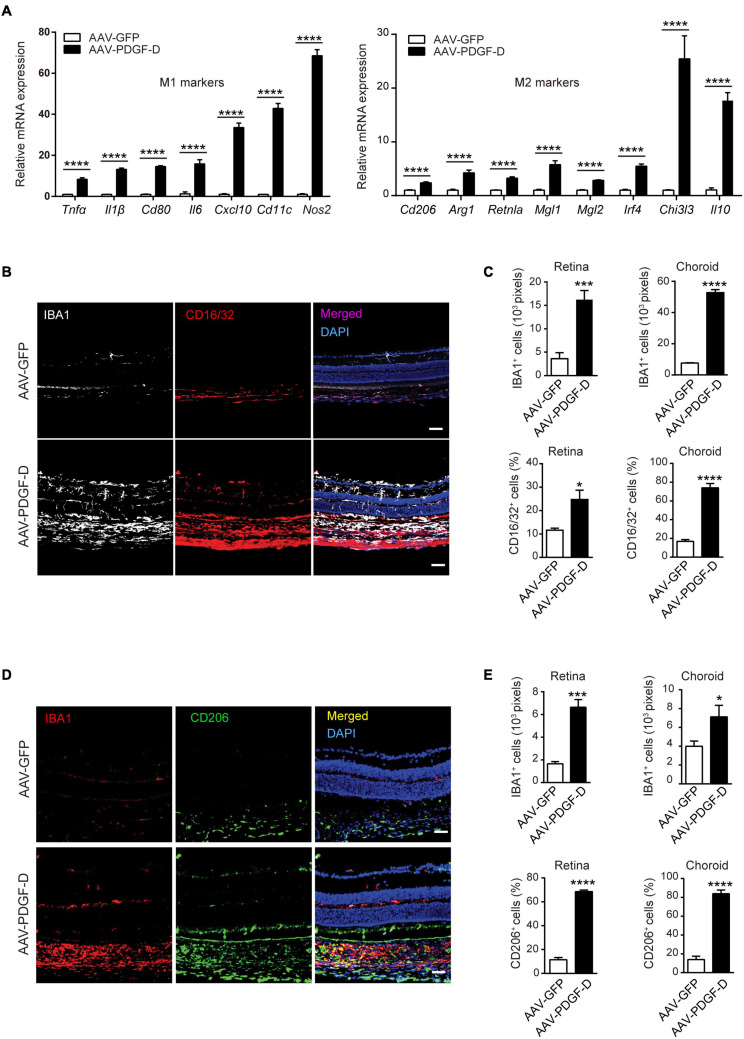
PDGF-D induced macrophage polarizations. **(A)** Real-time PCR analysis of markers of M1 and M2 macrophage polarization regulated by PDGF-D in mouse retinal pigment epithelium. **(B)** Immunofluorescence staining for IBA1^+^ and CD16/32^+^ (M1 marker) cells in mouse retinae and choroids with AAV-GFP or AAV-PDGF-D injection. **(C)** Quantifications of IBA1^+^ and CD16/32^+^ macrophage densities in **(B)**. **(D)** Immunofluorescence staining of IBA1^+^ and CD206^+^ (M2 marker) cells in mouse retinae and choroids with AAV-GFP or AAV-PDGF-D injection. **(E)** Quantification of CD206^+^ macrophage cell density in retinae and choroids in **(D)**. Scale bar: 50 μm. *n* = 5, *p < 0.05, ***p < 0.001, ****p < 0.0001.

The complement component C1qa has been implicated in the promotion of the of M2 microphage polarization to mitigate tissue inflammation (Spivia et al., 2014), while complement anaphylatoxin C3a and C5a are acknowledged to promote tissue inflammation by activating monocytes and macrophages (Bohlson et al., 2014). Interestingly, PDGF-D overexpression upregulated both M1 polarization markers, such as *Tnfα*, *Il1β*, *Nos2* and *Cxcl10*, and M2 polarization markers, such as *Arg1*, *Il10*, and *Chi3i3* (Figure 4A). Furthermore, immunofluorescence staining revealed that PDGF-D promoted IBA1^+^ macrophages in both retinae and choroids, which were also positive for CD16/32 staining, an M1 polarization marker (Figure 4B), with higher percentages in the PDGF-D overexpressing retinae and choroids (Figure 4C). In addition, IBA1^+^ macrophages were positive for CD206 staining, an M2 polarization marker (Figure 4D), with higher proportions in PDGF-D overexpressing choroids and retinae (Figure 4E). Together, these findings underline the presence of both pro- and anti-inflammatory milieu triggered by PDGF-D overexpression.

### PDGF-D Overexpression Increases Blood Vessel Density and Mural Cell Coverage

**FIGURE 5:**
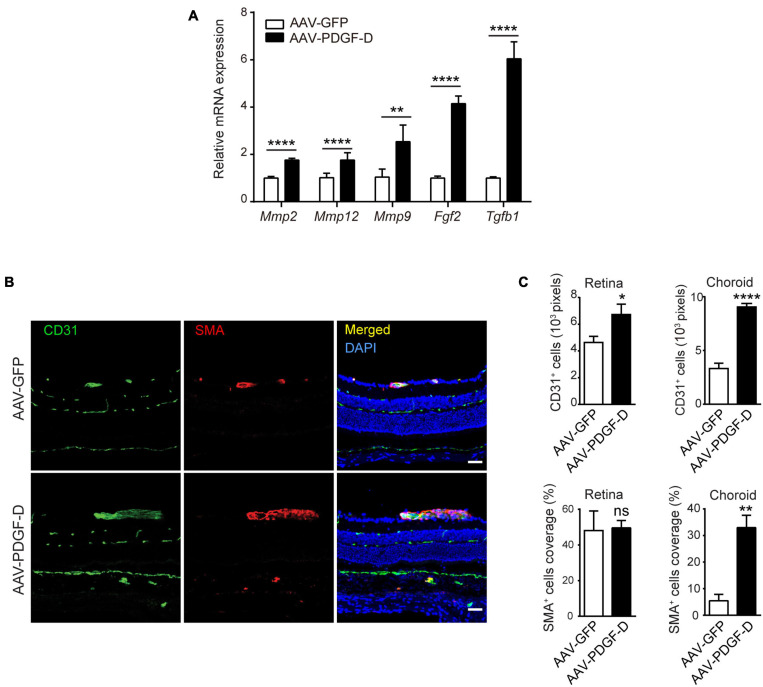
PDGF-D overexpression increased vascular endothelial cell density and mural cell coverage in mouse retinae and choroids. **(A)** Real-time PCR results showing angiogenic genes upregulated by PDGF-D overexpression in RPE-choroid complex. **(B)** Immunofluorescence staining of CD31^+^ endothelial cells (EC) and α-SMA ^+^ smooth muscle cells (SMC) in mouse retinae and choroids injected with AAV-GFP or AAV-PDGF-D. **(C)** Quantifications of CD31^+^ cell density and SMC coverage in retinae and choroids in **(B)**. Scale bar: 50 μm. *n* = 5, *p < 0.05, **p < 0.01, ****p < 0.0001, ns: not significant.

Neural retina is supported by the inner retinal blood vessels and outer retinal choriocapillaris beneath the RPE layer and Bruch membrane, which interacts with retinal microglia (McMenamin et al., 2019) and choroidal macrophages (Kumar et al., 2014). Since PDGF-D induced marked macrophage activation, we examined whether this affected retinal and choroidal blood vessel. In the PDGF-D overexpressing RPE-choroid complex, mRNA levels of genes encoding proangiogenic and extracellular matrix (ECM) regulators, such as *Tgfβ1*, *Fgf2*, *Mmp9*, *Mmp12* and *Mmp2*, were upregulated (Figure 5A). The PDGF family members are known to promote proliferation and recruitment of mural cells (Li et al., 2003; Uutela et al., 2004). PDGF-D overexpression increased CD31^+^ endothelial cell density in both retinae and choroids, while αSMA^+^ smooth muscle cell coverage increased only in choroids (Figures 5B,C). Thus, increased PDGF-D expression levels promoted retinal and choroidal blood vessel growth and maturation.

### Inflammatory Pathological Angiogenesis Triggered by PDGF-D

**FIGURE 6:**
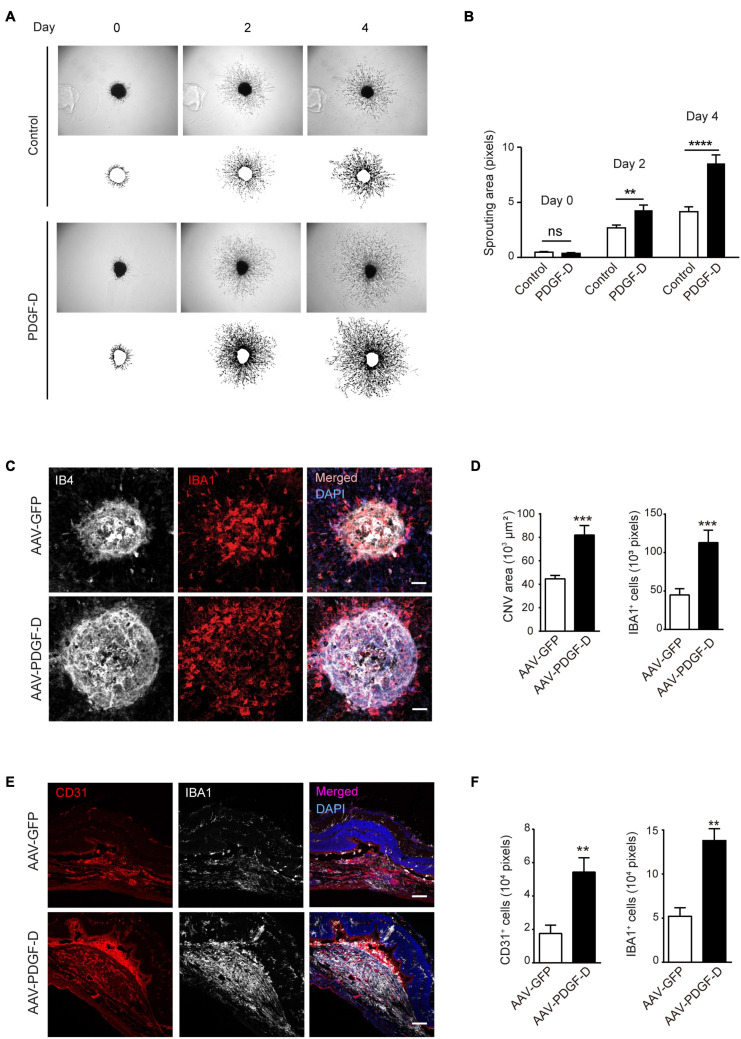
PDGF-D promotes choroid sprouting and pathological choroidal neovascularization (CNV).**(A)** PDGF-D protein treatment increased mouse choroidal sprouting at different days. **(B)** Quantifications of choroidal sprouting areas in **(A)**. **(C)** RPE-choroid wholemount immunofluorescence staining of IB4^+^ neovessels and IBA1^+^ macrophages in laser-induced CNVs with AAV-GFP or AAV-PDGF-D overexpression. **(D)** Quantifications of neovascular areas and IBA1^+^ macrophages in **(C)**. **(E)** Immunofluorescence staining of CD31^+^ ECs and IBA1^+^ macrophages in laser-induced CNVs. **(F)** Quantifications of the CD31^+^ ECs and IBA1^+^ macrophages in e. Scale bar: 50 μm. *n* = 5, **p < 0.01, ***p < 0.001, ****p < 0.0001, ns: not significant.

Choroidal blood vessels and RPE cells possess a unique symbiotic relationship by nourishing each other. The disruption of this association results in RPE degeneration or choroidal neovascularization (Lutty et al., 1999). Since PDGF-D overexpression increased choroidal endothelial cell density, we tested the effect of PDGF-D on choroids by stimulating choroidal explants with PDGF-D. PDGF-D promoted robust choroidal endothelial cell sprouting (Figures 6A,B) in a dose-dependent manner (Supplementary Figures 5A,B). Moreover, injury of the PDGF-D-overexpressing RPE cells by laser treatment augmented abnormal growth of IB4^+^ neovessels with more IBA1^+^ macrophages (Figures 6C,D). Furthermore, CD31^+^ pathological neovessels intermingled with IBA1^+^ macrophages (Figures 6E,F). These data indicated that the inflammatory milieu elicited by PDGF-D nurtured pathological choroidal neovascularization.

### Pharmacological Inhibition of the Complement Cascade Alleviates Inflammation and Pathological Neovascularization

**FIGURE 7:**
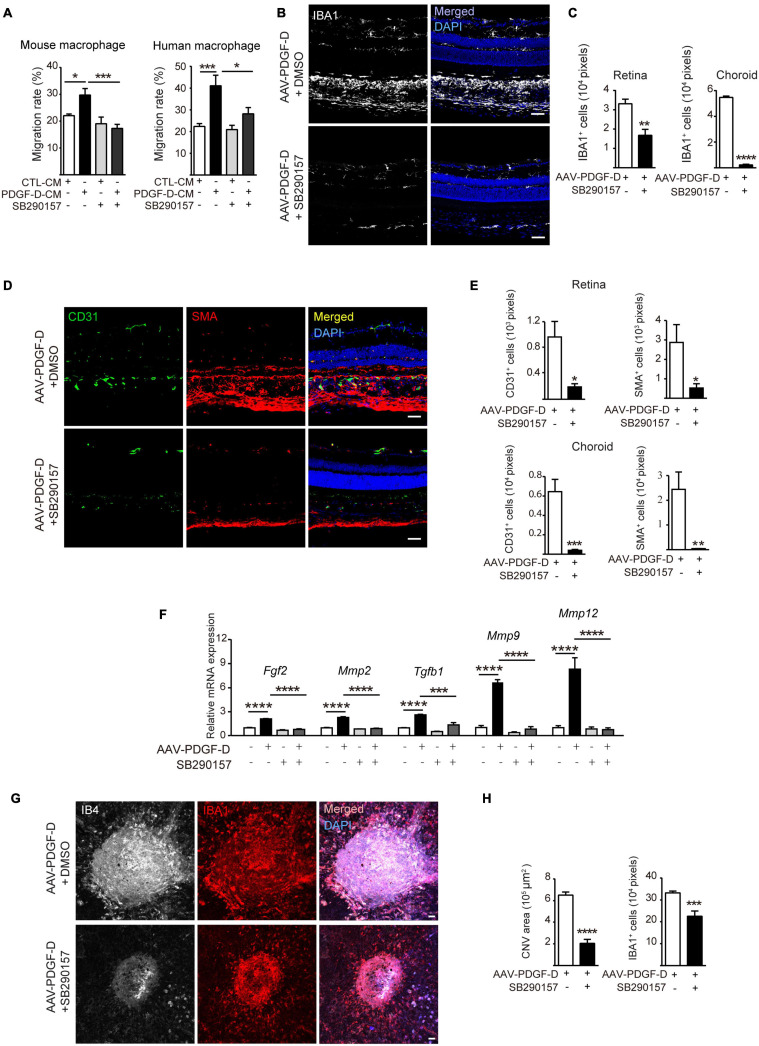
Inhibiting complement C3 cascade by SB290157 alleviates PDGF-D-induced inflammation and pathological neovascularization. **(A)** SB290157 (20 μM) inhibited migration of mouse and human macrophages treated with conditioned medium from PDGF-D-treated HRPE cells (PDGF-D-CM) for 24 h. Conditioned medium from HRPE cells without PDGF-D treatment (CTL-CM) was used as a control. **(B)** Immunofluorescence staining of IBA1^+^ macrophages in PDGF-D-overexpressing RPE-choroids treated with or without SB290157. **(C)** Quantification of IBA1^+^ macrophages in retinae and choroids in **(B)**. **(D)** Immunofluorescence staining of CD31^+^ ECs, NG2^+^ pericytes and α-SMA^+^ SMCs in PDGF-D-overexpressing RPE-choroids treated with or without SB290157. **(E)** Quantification of the CD31^+^, SMA^+^, and NG2^+^ cells in retinae and choroids in **(D)**. **(F)** Real-time PCR results showing that SB290157 inhibited PDGF-D-induced upregulation of angiogenic genes. **(G)** RPE-choroid wholemount immunofluorescence staining showing SB290157 reduced IB4^+^ neovessels and IBA1^+^ macrophages in laser-induced CNVs with AAV-GFP or AAV-PDGF-D overexpression. **(H)** Quantification of IB4^+^ and IBA1^+^ cells in **(G)**. Scale bar: 50 μm. *n* = 6, *p < 0.05, **p < 0.01, ***p < 0.001, ****p < 0.0001.

Since the induction and activation of the complement pathway by PDGF-D overexpression led to more macrophages and higher blood vessel density in the retinae and choroids, we tested whether blocking complement activation could inhibit pathological neovascularization and treated PDGF-D-stimulated macrophages and RPE with a C3a-receptor antagonist SB290157, which is known to block complement activation (Hutamekalin et al., 2010). SB290157 treatment inhibited migration of mouse and human macrophages (Figure 7A and Supplementary Figure 6A) *in vitro*. Furthermore, intraperitoneal injection of SB290157 to the RPE-specific PDGF-D overexpressing mice markedly reduced infiltration of IBA1^+^ macrophages in the retinae and choroids (Figures 7B,C) and suppressed the expression of both types of macrophage polarization markers (Supplementary Figure 6B). Additionally, immunofluorescence staining of endothelial and mural cells showed a marked reduction in blood vessel density and smooth muscle cell coverage (Figures 7D,E) with concomitant reduction of angiogenic and chemokine gene signatures (Figure 7F and Supplementary Figure 6C). Moreover, analysis of flat-mounted retinas confirmed the above findings by showing that PDGF-D overexpression increased retinal vascular branch points, which was abolished by SB290157 treatment (Supplementary Figures 7A,B). Since complement components are found in CNV lesions of AMD patients, the complement pathway was inactivated by SB290157 treatment in a mouse CNV model. Importantly, intraperitoneal injection of SB290157 inhibited CNV by reducing immune cell density in neovascularization lesions (Figures 7G,H). Together, these observations suggested that PDGF-D-induced activation of the complement pathway is critical for the promotion of macrophage infiltration and CNV formation.

## Discussion

The pathogenesis of AMD is associated with degenerative conditions in the neural retina, and dysfunctional RPE cells or choroids. A growing body of evidence has demonstrated that many inflammatory signals, such as chemokines, cytokines, growth factors and alterations in the complement pathway, can modify the functions of neuronal, vascular, glial, and immune cells to promote macular degeneration and pathological neovascularization. Our current work, for the first time, provides evidence that increased PDGF-D expression activated complement pathway to orchestrate tissue milieu by altering the expression of chemokines and cytokines responses to initiate macrophage activation and triggering neuroinflammatory conditions that exacerbate the pathogenesis of AMD.

The migration and activation of infiltrating inflammatory macrophages is detrimental to tissue function. Apart from exogenous stimuli, endogenously secreted molecules, such as cytokines, chemokines and growth factors, are essential to initiate macrophage migration (Lu et al., 1998; Apte, 2010; Qian et al., 2011; Jenkins et al., 2013; Gordon et al., 2014). PDGF-D expression in the synovial membranes of patients with rheumatoid arthritis and osteoarthritis is escorted with accumulation of synovial fibroblasts and macrophages (Pohlers et al., 2006). In chronic atherosclerotic lesions, PDGF-D was found to be colocalized with macrophages and promoted migration of THP1 monocytes in a dose-dependent manner (Wågsäter et al., 2009). Intriguingly, in our work, PDGF-D failed to promote migration of mouse or human macrophages directly. While we do not know the exact reasons for this discrepancy, it could be attributed to different experimental conditions. However, intriguingly, while PDGF-D did not promote macrophage migration by itself, conditioned medium from PDGF-D-stimulated RPE cells did promote the migration of mouse and human macrophages. Since PDGF-D is a potent stimulant of RPE proliferation and migration (Li et al., 2007; Kumar and Li, 2018), and RPE can secrete a wide array of immunomodulatory cytokines (Holtkamp et al., 2001) and chemokines (Ma et al., 2009) that can regulate macrophage response, PDGF-D-induced RPE secretome hence can promote the migration and activation of macrophages.

Over the last decade, mounting evidence has shown that the complement system plays an important role in the pathogenesis of AMD (Sparrow et al., 2012). With the initial finding of complement factor H (CFH) as a high-risk factor for AMD, additional studies identified C3a, C5a and the membrane-attacking complex C5b-9 in the drusen of AMD patients (Nozaki et al., 2006; Mullins et al., 2014). Indeed, transcriptomic analysis of PDGF-D-overexpressing RPE-choroid samples indicated that the presence of classical complement component C1qa and its activated product C3a. C1q expression was strongly associated with RPE cells and IA/IE^+^ macrophages, and C3a protein expression was seen in both the retinae and choroids. Interestingly, it has been shown that inhibition of PDGF-D using the monoclonal antibody CR002 decreased C5b-9 deposition in an experimental glomerulonephritis model (Ostendorf et al., 2006), supporting a role of PDGF-D in the regulation of the complement system. Several reports have also shown that activation of the complement pathway and C5b-9 can regulate the expression of numerous chemokines and cytokines (Kilgore et al., 1996; Selvan et al., 1998; Risnes et al., 2003). Additionally, under inflammatory conditions, RPE cells can generate a myriad of cytokines that can activate macrophages (Holtkamp et al., 2001). Indeed, engagement of both the classical (C1qa) and alternative (C3) complement pathways by PDGF-D overexpression markedly increased the levels of numerous chemokines and cytokines, and triggered polarization of both M1 and M2 macrophages. In fact, C1qa can downregulate inflammasome activation and promote the polarization of inflammation-resolving M2-like macrophages to engulf atherogenic lipoproteins (Bohlson et al., 2014; Spivia et al., 2014). While enhanced C3a signaling facilitates M1 polarization to exacerbate renal interstitial fibrosis, C3 gene deletion increased neovascularization in a mouse retinopathy model (Langer et al., 2010; Cui et al., 2019). PDGF-D has been demonstrated to have pleiotropic effects on vascular and non-vascular cells to stimulate pathological angiogenesis (Li et al., 2003; Kumar et al., 2010). Here, our data highlight yet another VEGF-A-independent function of PDGF-D by regulating the complement system and immune cells to promote CNV formation. Thus, PDGF-D imparted its effects by modulating the complement pathway to polarize macrophages, thereby promoting pathological neovascularization.

Targeting the complement system has been shown to protect mice from accumulating inflammatory mononuclear phagocytes in the subretinal space to maintain tissue homeostasis (Calippe et al., 2017). Complement fragments C3a, C5a, and C5b-9 are generated from C3 during complement activation. Treatment with SB290157, a potent and selective C3a-receptor antagonist, suppressed inflammation by blocking macrophage activation in animal models (Ames et al., 2001; Lim et al., 2013; Rowley et al., 2020). Indeed, we also observed that SB290157 treatment constrained macrophage polarization and blocked infiltration of macrophages by suppressing the expression of various chemokines, cytokines and growth factors, thus decreasing inflammatory neovascularization in the eye.

Collectively, our data reveal that increased PDGF-D levels activate the complement pathway, subsequently leading to marked macrophage activation and inflammation, the key pathologies of neovascular AMD. Therapeutic strategies targeting PDGF-D signaling and complement-mediated inflammation may provide new possibilities for the treatment of neovascular diseases.

## Data Availability Statement

The authors confirm that the RNA-seq data supporting the findings of this study are accessible in the GEO repository (Accession Number: GSE164972).

## Ethics Statement

The animal study was reviewed and approved by the Zhongshan Ophthalmic Center at the Sun Yat-sen University, Guangzhou, People’s Republic of China.

## Author Contributions

ZX and QW designed and performed the experiments, analyzed the data, and wrote a part of the manuscript. WL, LH, JiZ, and JuZ performed the experiments and analyzed the data. BX, SW, HK, XCL, and CL provided critical experimental tools and suggestions. XRL and AK designed the experiments, provided resources and supervision, analyzed the data, and wrote the manuscript. All authors contributed to the article and approved the submitted version.

## Conflict of Interest

The authors declare that the research was conducted in the absence of any commercial or financial relationships that could be construed as a potential conflict of interest. The handling editor declared a shared affiliation with the authors at the time of review.

## Supplementary Material

Supplementary Figure 1 PDGF-D does not affect macrophage proliferation. **(A)** Real-time PCR results showing expression of *Pdgfrα*, *Pdgfrβ*, and *Nrp1* in RAW264.7 mouse macrophages. **(B)** Real-time PCR results showing expression of *PDGFRα*, *PDGFRβ*,and *NRP1* in THP1 human monocytes and differentiated dTHP1 human macrophages. **(C,D)** PDGF-D protein treatment at different concentrations did not affect proliferation of mouse **(C)** or human **(D)** macrophages at 12 or 24 h. **(E,F)** PDGF-D protein treatment at different concentrations did not affect migration of mouse **(E)** or human **(F)** macrophages at 24 h. **(G,H)** Proliferation of mouse **(G)** and human **(H)** macrophages treated with conditioned medium from PDGF-D-treated HRPE cells (PDGF-D-CM). Scale bar: 400 μm. All the experiments were performed in triplicates, *****p* < 0.0001, ns: not significant.

Supplementary Figure 2 PDGF-D-induced RPE secretome promotes macrophage migration. **(A)** Immunoblot showing the activation of PDGFR-β by PDGF-D in HRPE. **(B,C)** Migration of mouse **(B)** and human **(C)** macrophages treated with conditioned medium from PDGF-D-treated HRPE cells (PDGF-D-CM). Scale bar: 400 μm. All the experiments were performed in triplicates. **p* < 0.05, ***p* < 0.01, ****p* < 0.001.

Supplementary Figure 3 Expression of PDGF-D receptors in mouse retina and choroid. **(A)** Real-time PCR results showing expression of PDGF receptors in retinae and RPE-choroid complex of normal C57BL6 mice. **(B)** Immunoblot analysis showing PDGF receptor expression in normal mouse retinae and RPE-choroid. **(C)** Immunofluorescence staining reveling PDGF receptor expression in normal mouse retinae and choroids. Scale bar: 50 μm. *n* = 5, **p* < 0.05, ***p* < 0.01, ns, not significant.

Supplementary Figure 4 RNA-seq and transcriptomic analysis showing PDGF-D induced. complement pathway, chemokine and cytokine signaling, extracellular matrix and growth factors. **(A,B)** Real-time PCR results showing PDGF-D-induced upregulation of complement pathway genes **(A)** and chemokine and their receptors **(B)** in mouse RPE-choroids. **(C,D)** Heatmaps of PDGF-D-induced DEGs associated to cytokine family **(C)** and extracellular matrix (ECM) and growth factors **(D)** n mouse RPE-choroids. **(E,F)** Real-time PCR results showing PDGF-D-induced upregulation of Il7r **(E)** and extracellular matrix genes **(F)**. *n* = 5, **p* < 0.05, ****p* < 0.001, *****p* < 0.0001.

Supplementary Figure 5 PDGF-D promotes mouse choroid sprouting in a dose-dependent manner. **(A)** PDGF-D protein treatment induced mouse choroidal sprouting. **(B)** Quantifications of the choroidal sprouting in **(A)**. *n* = 5, ***p* < 0.01, ****p* < 0.001, *****p* < 0.0001, ns: not significant.

Supplementary Figure 6 SB290157 inhibits PDGF-D-induced macrophage migration and gene expression. **(A)** SB290157 inhibited mouse (upper penal) and human (lower panel) macrophage migration induced by conditioned medium from PDGF-D-treated HRPE cells (PDGF-D-CM). **(B,C)** Real-time PCR results showing that SB290157 inhibited AAV-PDGF-D-induced upregulation of M1 and M2 macrophage polarization genes **(B)** and cytokine and chemokine genes **(C)**. Scale bar: 500 μm. All the experiments were performed in triplicates, ***p* < 0.01, ****p* < 0.001, *****p* < 0.0001.

Supplementary Figure 7 Vascular changes in the flat-mounted retinas with PDGF-D overexpression with or without SB290157 treatment. **(A)** Analysis of flat-mounted retinas after IB4 staining (green) showing that PDGF-D overexpression increased retinal vascular branch points, which was abolished by SB290157 treatment. **(B)** Quantifications of vascular branch points in the mouse retinas with PDGF-D overexpression with or without SB290157 treatment. Scale bars in **(A)**: upper panel 500 μm, lower panel 50 μm, *n* = 8, ****p* < 0.001.
