# Corrigendum: Platelet-Derived Growth Factor-D Activates Complement System to Propagate Macrophage Polarization and Neovascularization

**DOI:** 10.3389/fcell.2022.848292

**Published:** 2022-02-08

**Authors:** Zhen Xiong, Qianqian Wang, Wanhong Li, Lijuan Huang, Jianing Zhang, Juanhua Zhu, Bingbing Xie, Shasha Wang, Haiqing Kuang, Xianchai Lin, Chunsik Lee, Anil Kumar, Xuri Li

**Affiliations:** State Key Laboratory of Ophthalmology, Zhongshan Ophthalmic Center, Sun Yat-sen University, Guangzhou, China

**Keywords:** PDGF-D, C1qa, C3, macrophage polarization, inflammation

In the original article, there was an error. In typesetting, the texts under the subtitle *RNA Isolation and Real Time quantitative PCR* from the section **Materials and Methods** were accidentally overwritten by the content under the subtitle, *RNA Sequencing and Transcriptome Analysis*.

A correction has been made to **Materials and Methods**, *RNA Isolation and Real Time Quantitative PCR, Paragraph 1*.

“Total RNA was isolated using the TRNzol reagent (TIANGEN, cat: DP424) and converted to cDNA using the Fast King RT Kit (TIANGEN, cat: KR116) according to the manufacturer’s instructions. Real-time quantitative PCR was carried out in a 10 μl reaction containing the SYBR Select Master Mix (Vazyme, cat: Q331) in technical quadruplicate using a Quantstudio 6K Flex system (Life Technologies). Results were analyzed using the Quantstudio 12 K Flex Software v1.2.2 (Thermo Fisher Scientific). Relative mRNA levels were calculated based on the 2^−∆∆CT^ method, using the 18S rRNA as references.”

The authors apologize for this error and state that this does not change the scientific conclusions of the article in any way. The original article has been updated.

