# Supplementary figures and images for: Platelet-Derived Growth Factor-D Activates Complement System to Propagate Macrophage Polarization and Neovascularization
Source: Front Cell Dev Biol. 2021 Jun 2;9:686886. doi: 10.3389/fcell.2021.686886 (PMC8207142; doi:10.3389/fcell.2021.686886)

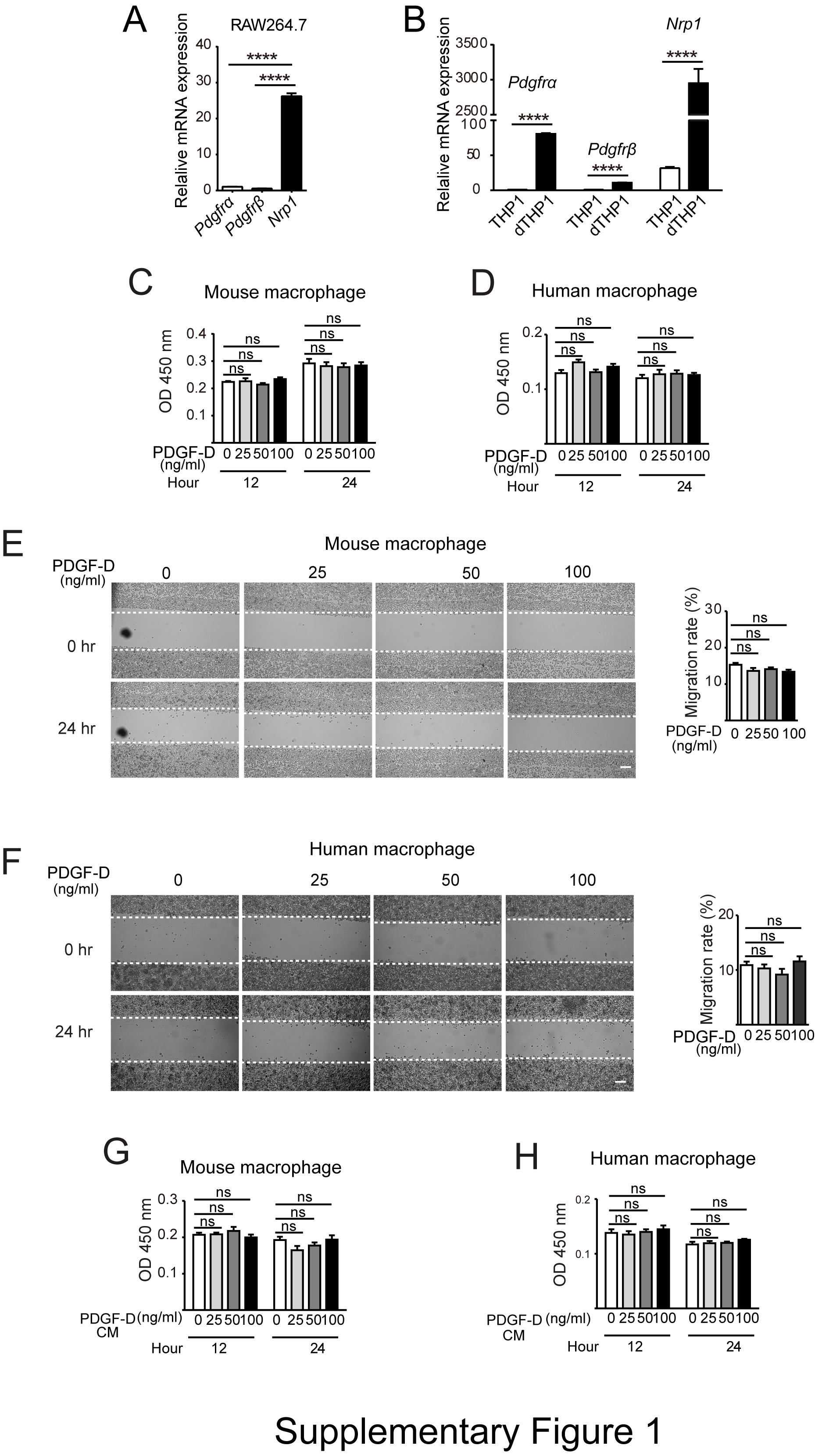

Supplement: Supplementary Figure 1 — PDGF-D does not affect macrophage proliferation. (A) Real-time PCR results showing expression of Pdgfrα, Pdgfrβ, and Nrp1 in RAW264.7 mouse macrophages. (B) Real-time PCR results showing expression of PDGFRα, PDGFRβ,and NRP1 in THP1 human monocytes and differentiated dTHP1 human macrophages. (C,D) PDGF-D protein treatment at different concentrations did not affect proliferation of mouse (C) or human (D) macrophages at 12 or 24 h. (E,F) PDGF-D protein treatment at different concentrations did not affect migration of mouse (E) or human (F) macrophages at 24 h. (G,H) Proliferation of mouse (G) and human (H) macrophages treated with conditioned medium from PDGF-D-treated HRPE cells (PDGF-D-CM). Scale bar: 400 μm. All the experiments were performed in triplicates, ****p < 0.0001, ns: not significant. [file Image_1.TIF]

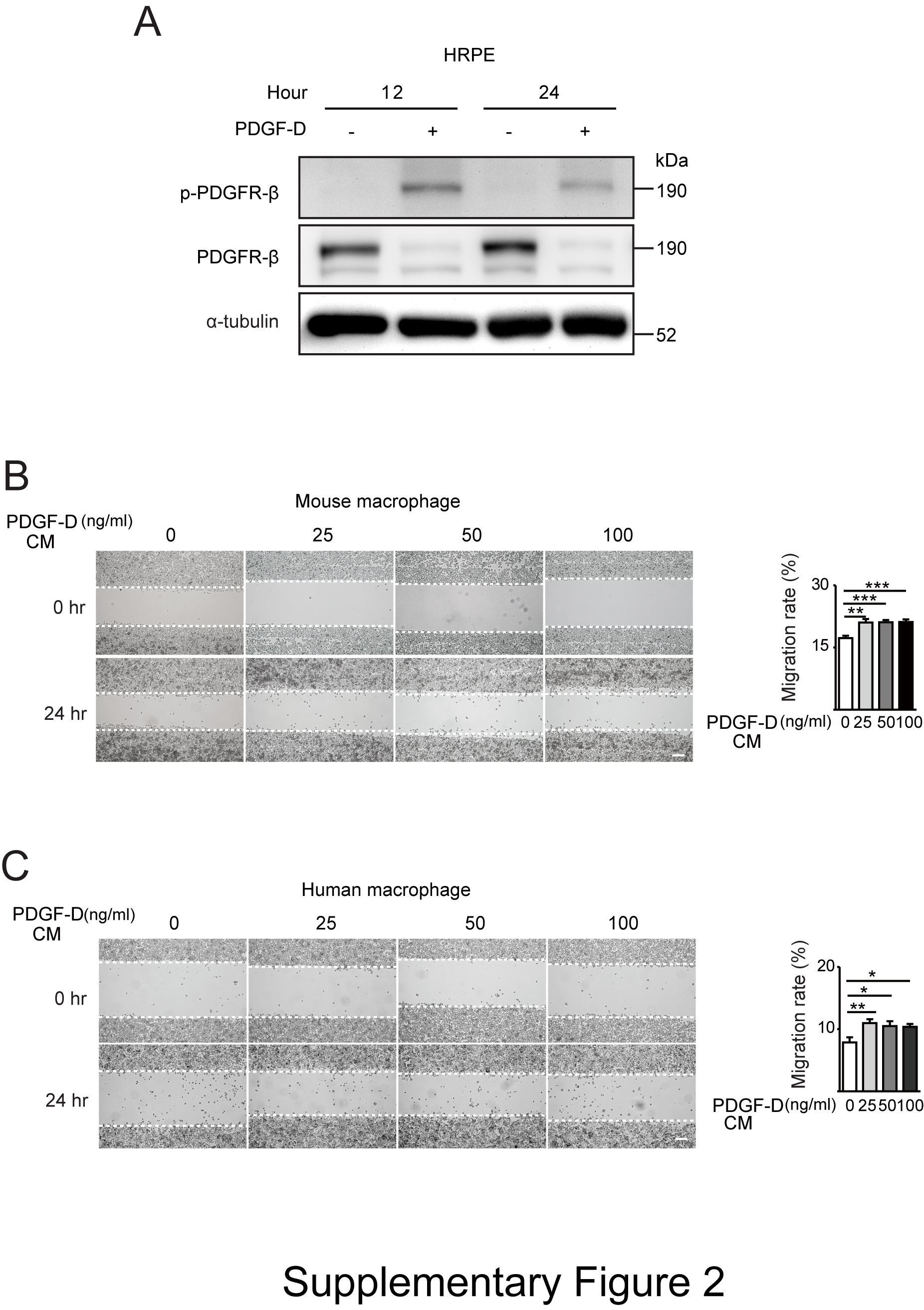

Supplement: Supplementary Figure 2 — PDGF-D-induced RPE secretome promotes macrophage migration. (A) Immunoblot showing the activation of PDGFR-β by PDGF-D in HRPE. (B,C) Migration of mouse (B) and human (C) macrophages treated with conditioned medium from PDGF-D-treated HRPE cells (PDGF-D-CM). Scale bar: 400 μm. All the experiments were performed in triplicates. *p < 0.05, **p < 0.01, ***p < 0.001. [file Image_2.TIF]

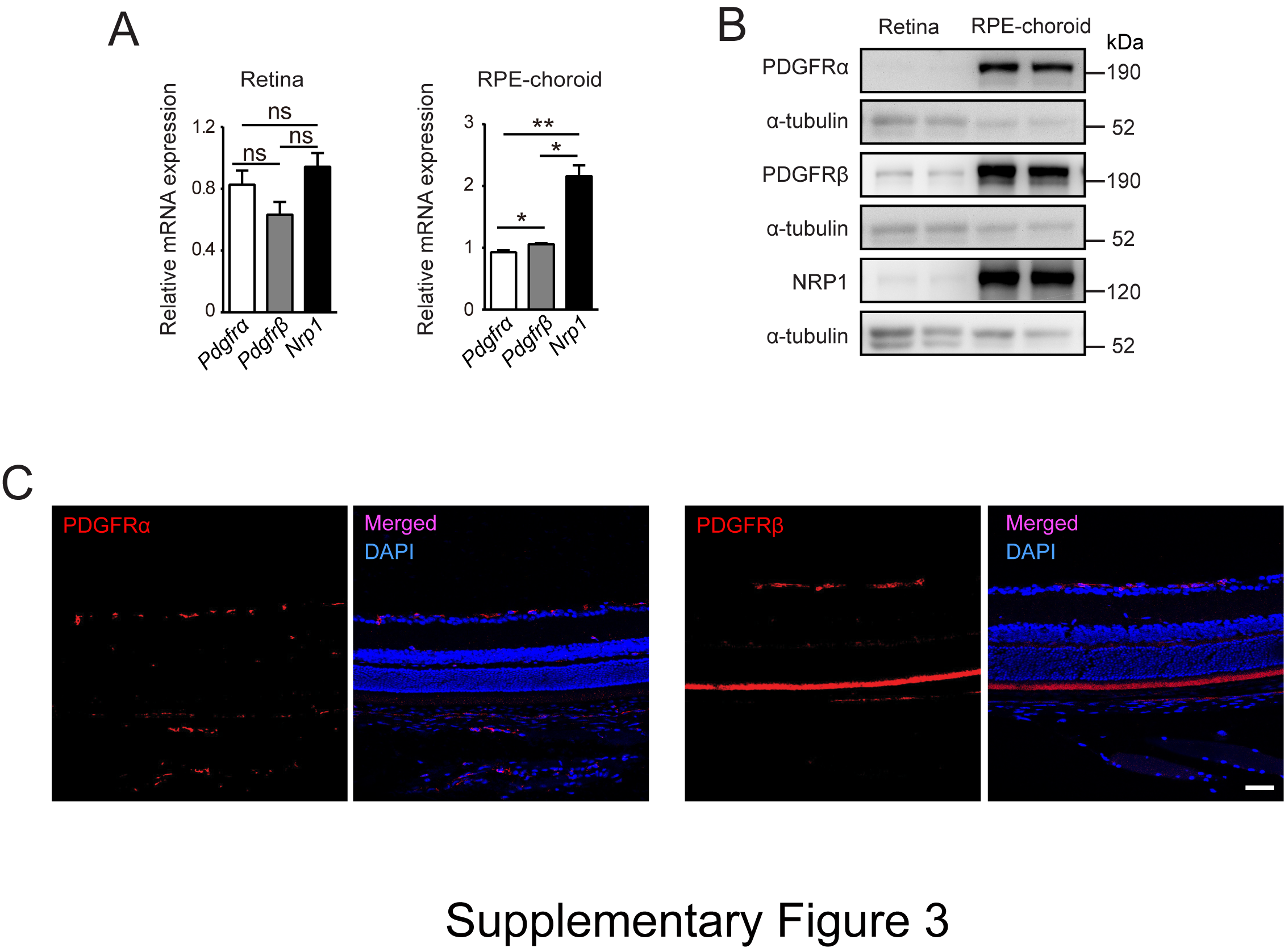

Supplement: Supplementary Figure 3 — Expression of PDGF-D receptors in mouse retina and choroid. (A) Real-time PCR results showing expression of PDGF receptors in retinae and RPE-choroid complex of normal C57BL6 mice. (B) Immunoblot analysis showing PDGF receptor expression in normal mouse retinae and RPE-choroid. (C) Immunofluorescence staining reveling PDGF receptor expression in normal mouse retinae and choroids. Scale bar: 50 μm. n = 5, *p < 0.05, **p < 0.01, ns, not significant. [file Image_3.TIF]

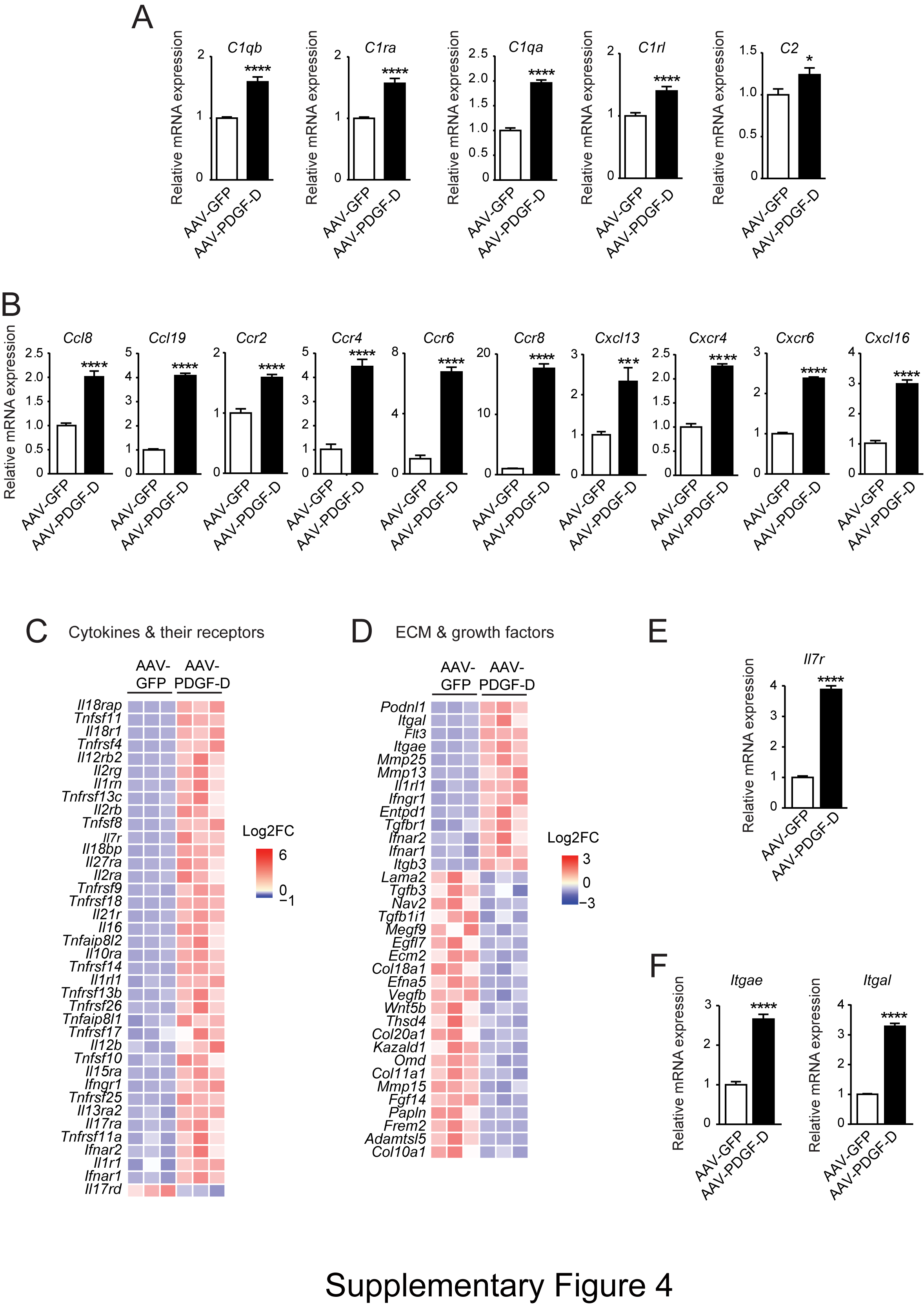

Supplement: Supplementary Figure 4 — RNA-seq and transcriptomic analysis showing PDGF-D induced. complement pathway, chemokine and cytokine signaling, extracellular matrix and growth factors. (A,B) Real-time PCR results showing PDGF-D-induced upregulation of complement pathway genes (A) and chemokine and their receptors (B) in mouse RPE-choroids. (C,D) Heatmaps of PDGF-D-induced DEGs associated to cytokine family (C) and extracellular matrix (ECM) and growth factors (D) n mouse RPE-choroids. (E,F) Real-time PCR results showing PDGF-D-induced upregulation of Il7r (E) and extracellular matrix genes (F). n = 5, *p < 0.05, ***p < 0.001, ****p < 0.0001. [file Image_4.TIF]

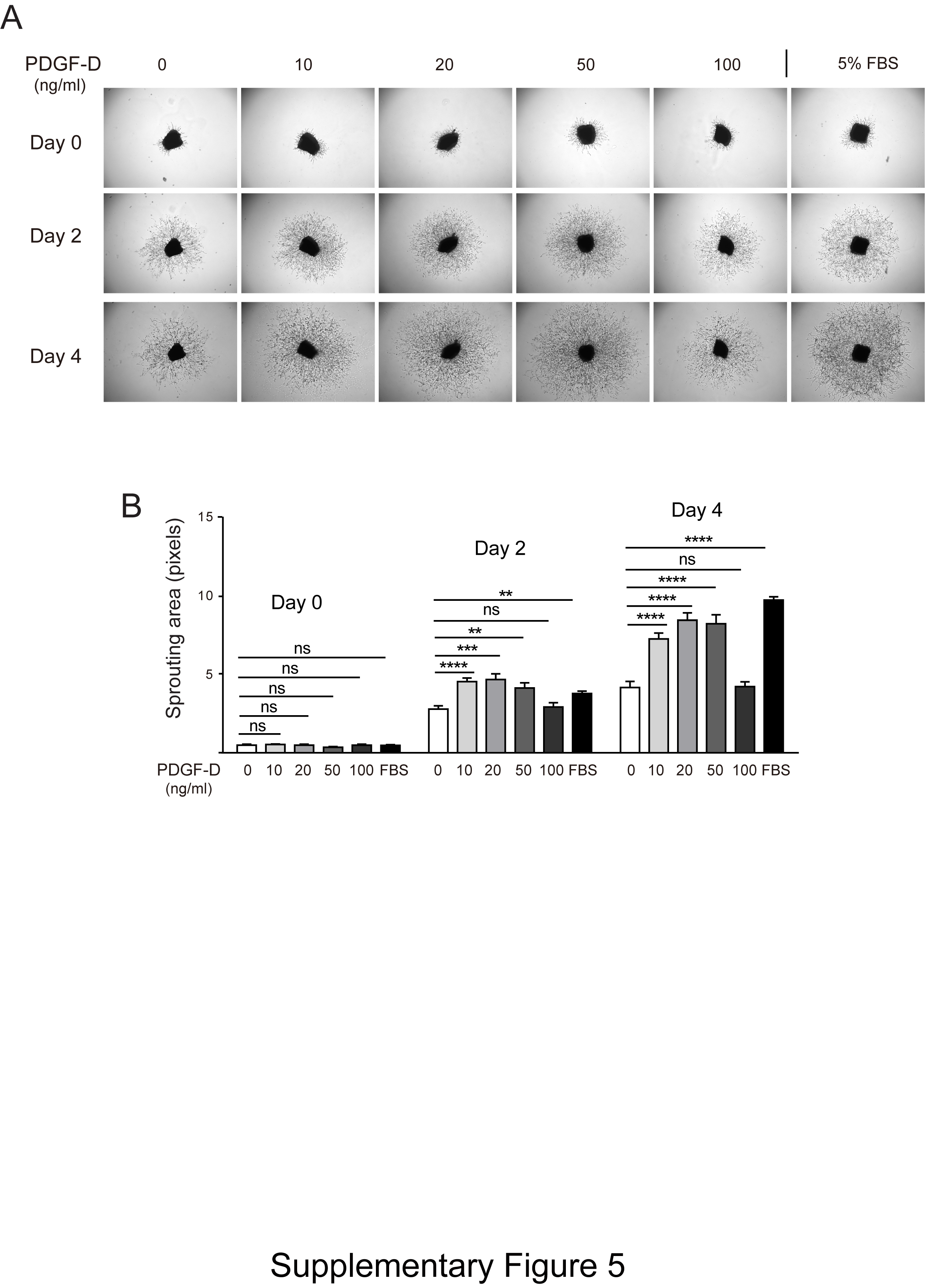

Supplement: Supplementary Figure 5 — PDGF-D promotes mouse choroid sprouting in a dose-dependent manner. (A) PDGF-D protein treatment induced mouse choroidal sprouting. (B) Quantifications of the choroidal sprouting in (A). n = 5, **p < 0.01, ***p < 0.001, ****p < 0.0001, ns: not significant. [file Image_5.TIF]

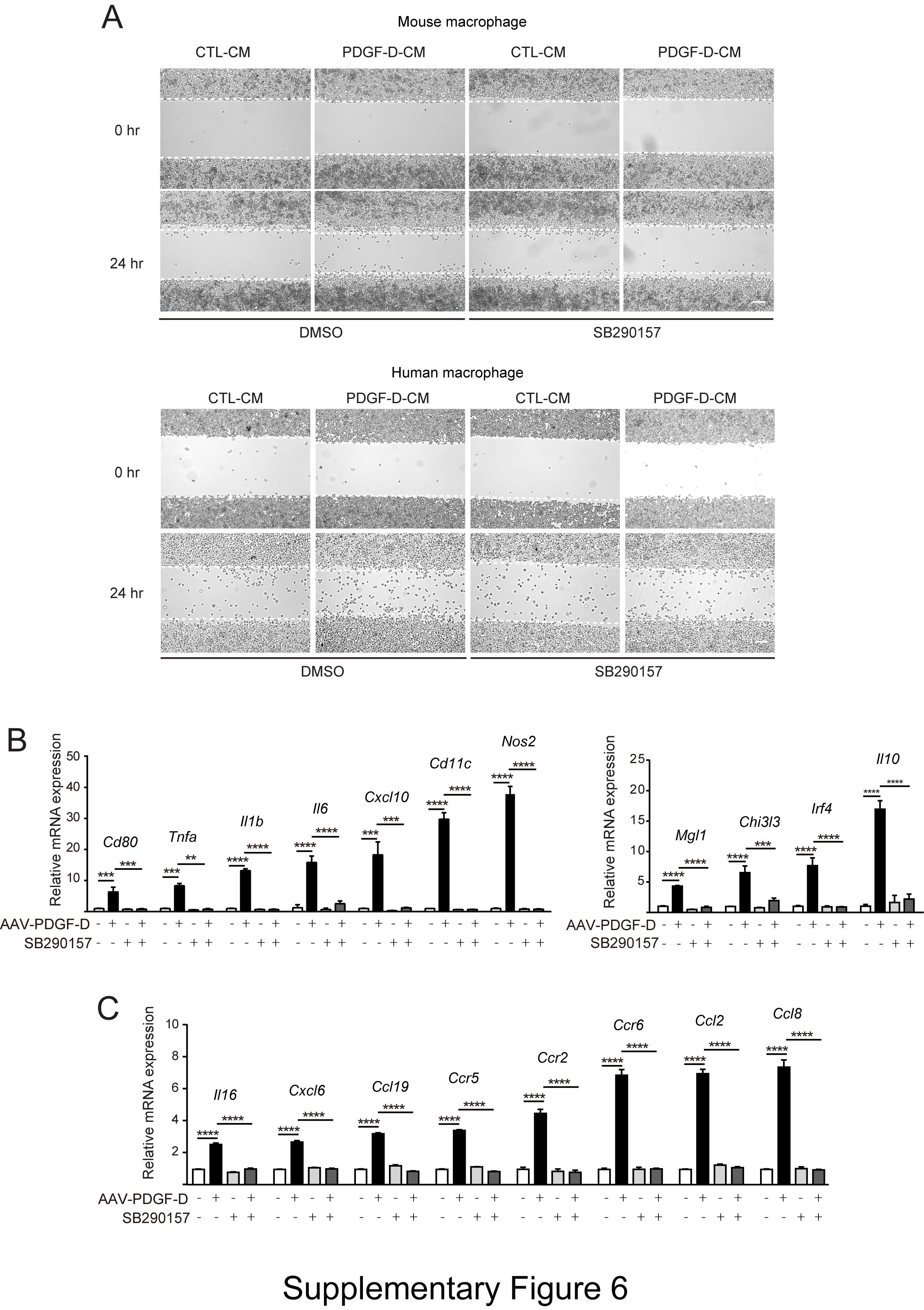

Supplement: Supplementary Figure 6 — SB290157 inhibits PDGF-D-induced macrophage migration and gene expression. (A) SB290157 inhibited mouse (upper penal) and human (lower panel) macrophage migration induced by conditioned medium from PDGF-D-treated HRPE cells (PDGF-D-CM). (B,C) Real-time PCR results showing that SB290157 inhibited AAV-PDGF-D-induced upregulation of M1 and M2 macrophage polarization genes (B) and cytokine and chemokine genes (C). Scale bar: 500 μm. All the experiments were performed in triplicates, **p < 0.01, ***p < 0.001, ****p < 0.0001. [file Image_6.TIF]

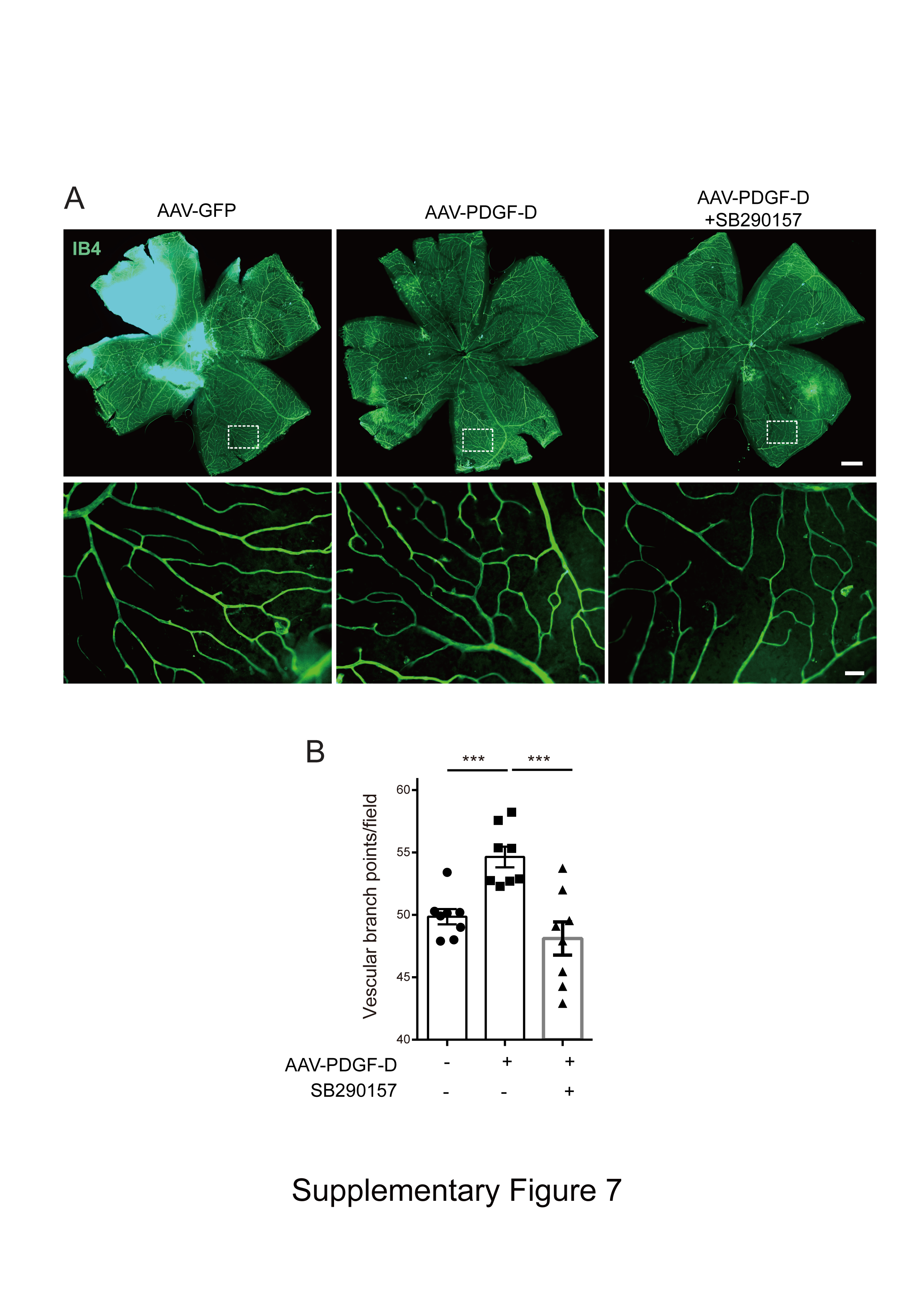

Supplement: Supplementary Figure 7 — Vascular changes in the flat-mounted retinas with PDGF-D overexpression with or without SB290157 treatment. (A) Analysis of flat-mounted retinas after IB4 staining (green) showing that PDGF-D overexpression increased retinal vascular branch points, which was abolished by SB290157 treatment. (B) Quantifications of vascular branch points in the mouse retinas with PDGF-D overexpression with or without SB290157 treatment. Scale bars in (A): upper panel 500 μm, lower panel 50 μm, n = 8, ***p < 0.001. [file Image_7.TIF]
